# Collaborative diabetes training in outpatient primary care

**DOI:** 10.1080/21614083.2017.1288490

**Published:** 2017-02-23

**Authors:** Tiina Tervaskanto-Mäentausta, Anja Taanila, Olavi Ukkola, Leila Mikkilä, Jari Jokinen, Essi Varkki

**Affiliations:** ^a^ Center for Life Course Health Research, University of Oulu, Oulu, Finland; ^b^ Oulu University of Applied Sciences, Oulu, Finland; ^c^ Health centre of the City of Oulu, Oulu, Finland

**Keywords:** Primary care, type 2 diabetes, patient centredness, collaborative learning, medical and nurse education

## Abstract

Two Universities from Oulu, Finland organised integrated and interprofessional (IP) type 2 diabetes training periods for undergraduate medical and nursing students in collaboration with the University Hospital and Health and Wellbeing Centre of Oulu. There is an ongoing health, social services and regional government reform in Finland. The services will be organised in a customer-orientated way and the reform will combine the primary and secondary services. The training was tailored to reflect the real life future setting in Finnish primary care, and this model fits well with the principles of collaborative education. The study aimed at investigating students’ attitudes and readiness for inter professional learning and their learning experience in combined primary and secondary care settings. The second aim was to strengthen students’ professional skills by working with patients in a patient-centred manner. The “Readiness for Interprofessional Learning Scale” was used with added questions about pair training. Students’ perceptions of their clinical skills were evaluated. The students valued the mutual learning experience in outpatient primary care. They felt comfortable with working together and complemented each other. Students performed well with IP competencies such as patient centredness, communication and team functioning. Patients in general were very satisfied with the visit. Teamwork and collaboration, professional identity and pair work were highly scored in both student groups while roles and responsibilities were evaluated a little less positively. Collaboration between different levels of care and health policies is important when developing health professionals’ education. This IP teamwork experience helps both future and current health-care professionals to better organise the care of chronic illnesses.

## Introduction

There is an ongoing health, social services and regional government reform in Finland.[1] The aims of the reform will be to even out differences in health and wellbeing and to curb cost increases. Health and social services will be organised in a more customer-oriented way, and primary health and social services as well as specialised hospital care will be administered and funded by the same regional authority. The key objectives of the reform are to promote the health, wellbeing and social safety of the population, ensure equal access to services, and enhance primary care.

Diabetes type 2 is one of the most common chronic illnesses in Finland as well as in other developed countries. The importance of diabetes to public health is mainly based on the complications associated with the disease. People with diabetes run a significantly higher risk of cardiovascular diseases.[2] The city of Oulu is located in northern Finland and has about 200,000 inhabitants. It has been calculated that 10% of the citizens account for 81% of the social and health care expenditure.[3] Moreover, 11% of these heavy service users have diabetes and cardiovascular diseases.[3] The overall costs of diabetes and its complications have increased markedly.[2] A key component to improve better acute, chronic and preventive evidence based care and self-care support of the patient is to build effective interprofessional (IP) teams in primary health care.[4–6] In diabetes type 2 care patient centred collaboration between primary and specialised hospital services is essential. To our knowledge there are limited models of this form of collaboration in health care education.

This study is also significant because interprofessional education (IPE) at undergraduate level in primary care is less implemented and researched than IPE in hospital settings. Most previous research in primary health care has focused on professional level and strengthening IP teams.[7] Janson et al. [6] noted that IP teams consisting of primary care internal medicine residents, nurse practitioner students and pharmacy students were effective in improving quality of care for adult patients with diabetes treated in general medicine clinics, and further noted that chronic illness framework resulted in more appropriate health care utilisation.[6] Bunniss and Kelly [4] found out that experimental and shared learning motivated the professionals to prioritise patients’ needs and understand the roles and skills of each other. Delva et al. [5] discovered that clear goals and attention to teamwork at all levels of collaboration are needed if effective IPE is to be achieved.

Professional silos often hinder effective collaboration. There is evidence that IPE can help to break down stereotypical views that professionals hold about one another and can result in an increased understanding of the roles, responsibilities, strengths and limitations of other professions.[8,9] IPE is a process where students from at least two different educational backgrounds learn together, learn about each other, and learn from each other, during certain periods of their education.[9–11] Developing IPE and IP training (IPT) involves staff from different faculties, work settings and locations to work together.[11,12]

The Canadian framework of IP competencies includes such aspects as patient and family centred approaches, communication skills, team work skills, clarification of roles and responsibilities, IP conflict resolution skills and collaborative leadership skills.[13] There are increasingly complex issues in public health care, which no health or social profession is able to solve alone, thus all professionals need IP competencies. Furthermore, these increasingly complex issues need communication and collaboration also between specialised hospitals, primary care and higher education institutes in order to share expertise and develop future services.[7,14,15]

University of Oulu, Faculty of Medicine and Oulu University of Applied Sciences planned and organised integrated and interprofessional training (IPT) periods for undergraduate medical and nursing students in collaboration with the University Hospital and Health Centre of the city of Oulu. The longitudinal goal was to develop curricula which will help students to develop the knowledge, skills and abilities to train in IP team in primary care, and with patients suffering a chronic disease. Type 2 diabetes patients were chosen as a focus group based on the priorities of the national public health strategy of Finland. Hean pointed out that in order to develop IP competencies learners need to develop the ability to integrate knowledge, skills, attitudes, and values in making professional judgements.[16] This study aims at investigating students’ attitudes and readiness for interprofessional learning (IPL) and their learning experiences in combined primary and specialised hospital care settings. The second aim of this study is to find out if students’ professional skills are strengthened by working as an IP team with patients in a patient centred manner.

## Design of the IP training

First, the IP planning group was created. The participants were an endocrinologist from Oulu University Hospital, a general practitioner and a diabetes nurse from the Health centre of Oulu, a clinical teacher and an educational coordinator from University of Oulu, Faculty of Medicine, and a senior lecturer from Oulu University of Applied Sciences. Earlier good experience of IP training was the starting point.[17,18] Third-year medical students and second- or third-year nursing students participated in the IP training. One training period lasted half a day.

Two medical students and one nurse student planned and carried out together an outpatient visit of a patient with diabetes type 2. Two IP student groups were working simultaneously. Facilitators prepared pre material for the students, such as recommendations of the evidence based care and guidelines for patient examination. Health centre personnel gave general info to the students about practical issues in the health centre, and health recording system. Students familiarised themselves with the patient history and agreed on the team tasks. Student teams interviewed and examined the patients. They performed physical examination including RR, HR, BMI, and diabetic foot evaluation (monofilament, vibration tuning fork) as well as measured F-glucose. Student teams reviewed nutritional status and gave guidance to the patients. Student teams discussed with the facilitating doctor and nurse about the findings, and made treatment and care plan together. The visit was finalised with the patient, the team and the facilitators. Students entered the documentation into the electronic patient recording system. After the training all facilitators and students attended a reflective debriefing session and students gave structured feedback. The patients gave oral feedback and eight of them also written feedback. Altogether 93 students (medical *n* = 64, nurse *n* = 29) and 23 patients participated in the training in 2013–2015.

After the training students evaluated their attitudes towards IPL by using a modified “Readiness for Interprofessional Learning Scale” (RIPLS) [8] with added questions about pair training. Comparisons between the medical and nurse students were made. Students’ perceptions of their clinical skills were analysed in each statement.[19] The patients gave oral feedback of the visit and eight of them filled a structured questionnaire consisting of 14 statements of their experience with the scale 1–5 as well as positive and negative adjectives to describe the atmosphere during the visit.

The attitudes and readiness of the students for IPL were investigated using sum squares of the three subscales of RIPLS (teamwork and collaboration, professional identity, roles and responsibilities) presented by Parsell and Bligh [8]. In addition, pair work questions were analysed similarly. The differences between the groups (medical students and nursing students) were investigated by analysis of variance (ANOVA). The quantitative data was analysed using IBM SPSS Statistics, version 22 (1989, 2013 SPSS, Inc., Chicago, IL, USA, an IBM company). Open questions of the questionnaires were analysed by content analysis.[19]

## Results

**Figure 1.**
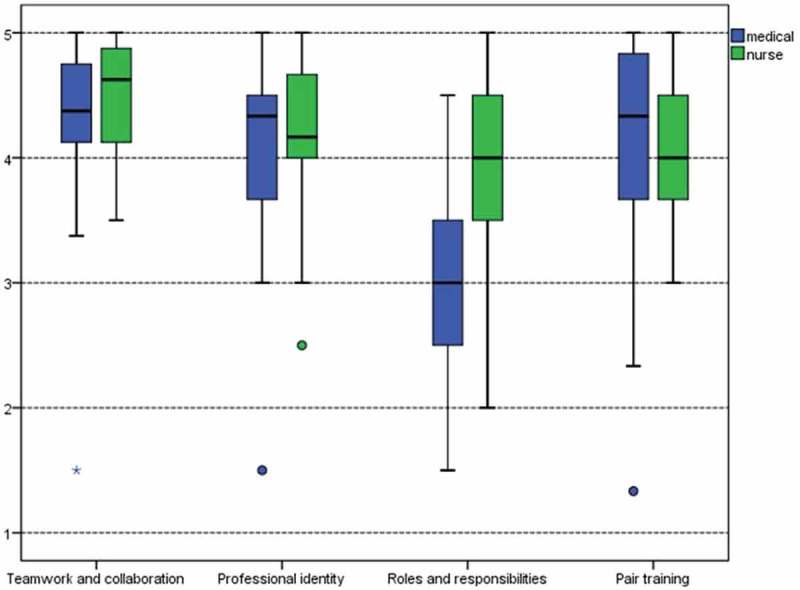
Students’ attitudes towards IPE and pair training (1 totally disagree to 5 totally agree).

“Teamwork and collaboration” was highly valued in both student groups (Figure 1). The perceptions of nursing (N) students (mean 4.361) were slightly stronger than medical (M) students (4.498; *p* = 0.353). Similar results were seen in “professional identity” (M 4.089, N 4.218; *p* = 0.348), and “pair work” (M 4.138, N 4.040; *p* = 0.311), but the tendency for these scales among medical students tended to be slightly stronger when compared to nursing students. “Roles and responsibilities” (M 3.032, N 3.810; *p* < .0005) were evaluated slightly lower than the other scales in both groups. Statistically significant differences between the groups were only seen in this subscale, and perceptions of medical students were lower than nursing students.

The statements were also examined separately. The most distinct statements were further analysed. The “teamwork and collaboration” subscale showed highly positive perceptions in several statements. Both medical and nursing students agreed strongly (68.1%) that it is for the patients’ best interest that professionals work as a team. Statement “learning with students from other programmes will help me to become a more effective member of a health care team” was agreed by 87% of both student groups. Statement “communication skills should be learnt with other health care students” was agreed by 78.7% of medical students and by 86.3% of nursing students. There was also strong agreement with the statements of the subscale “professional identity”. Statement “patients would ultimately benefit if health care students worked together to solve patient problems” was agreed by 80% of both student groups. Subscale “roles and responsibilities” showed that nursing students (60%) felt in a stronger way that their role is not to be only an assistant to the doctors when compared to medical students (40%). Statement “pair training clarified the overall view of preventive and holistic health care” was agreed by 78.1% of medical students and by 77.3% of nursing students.

**Figure 2.**
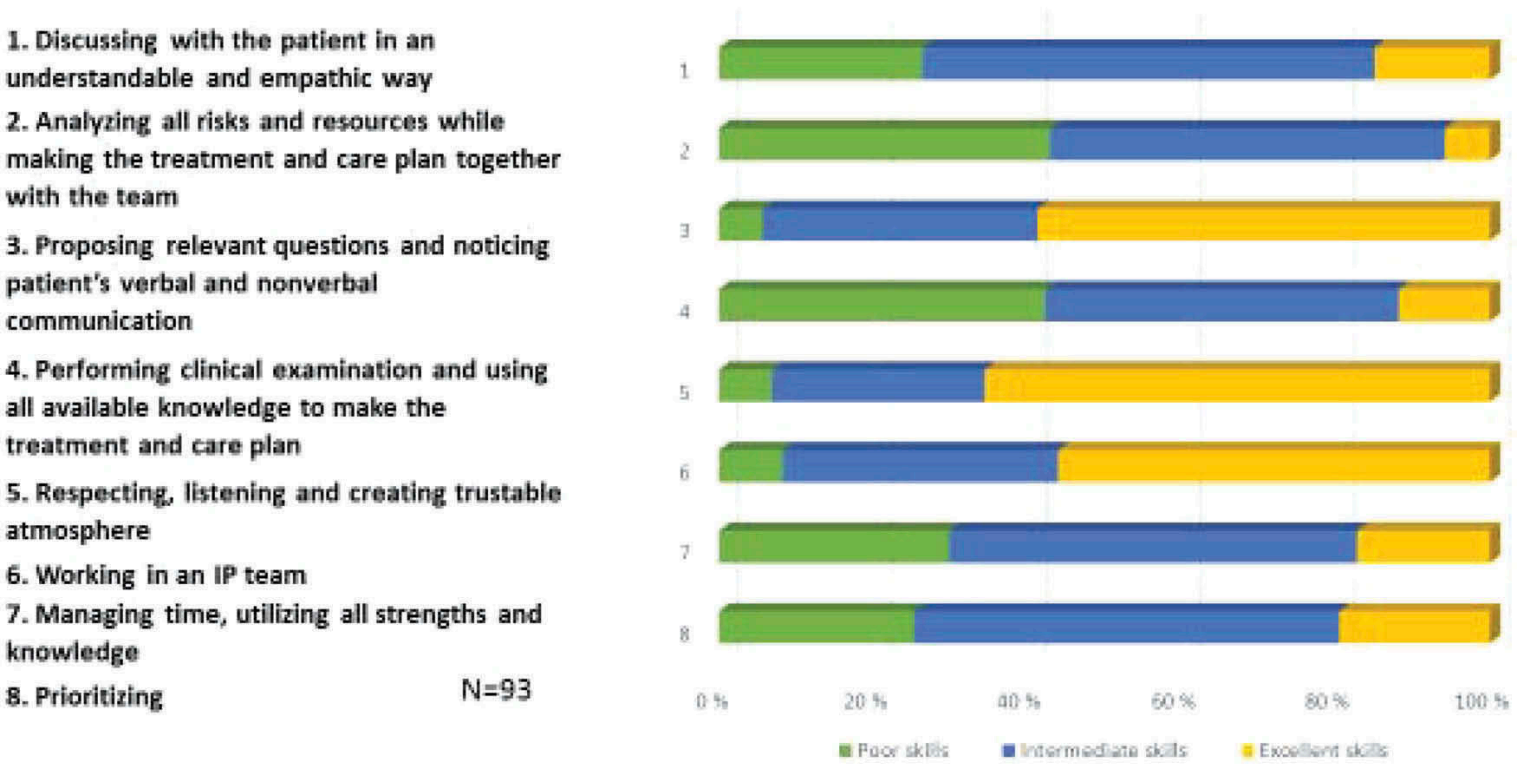
Students’ perception of their clinical skills (1 poor skills; 2 intermediate skills; 3 excellent skills).

Students’ perception of their clinical skills were analysed in eight statements with three options (poor skills, intermediate skills, excellent skills) (Figure 2). About 60% of the students evaluated their skills in proposing relevant questions and noticing patients’ nonverbal communication as excellent. Also about 60% of them evaluated skills in respecting, creating a trustable atmosphere and working in a team as excellent. About 40% of the students evaluated their skills as poor when analysing all risks and resources while making the care plan in a team, as well as when performing clinical examination, and making treatment and care plan.

The students commented in open questions about their learning experience. Most of them felt that team support made the situation more relaxed. While working in the team they complemented each other, and learned about the value of IP team work. They appreciated the holistic and patient centred approach during the visit. Almost all found diabetes patients educational, the tasks were clear and the facilitation was excellent. Some students struggled in the beginning in finding their role in the team and some wished they had more time to read the patient files before the visit. In debriefing sessions students commented that taking responsibility of the whole visit helped them to understand the “big picture” of the outpatient care of the diabetes type 2 patient in primary care, and the importance of collaboration between primary and specialised health care. Almost all wished they had more of this kind of learning and training possibilities.

In the first training periods patients gave oral feedback. From autumn 2015 the feedback was collected also by questionnaire and patients completed the questionnaire. In general patients were very satisfied with the visit. They felt that the atmosphere was trustable and patient centred, and the staff respected each other and worked professionally. Patients appreciated that they had enough time to be heard, and commented that the reality was much better than expected.

**Figure 3.**
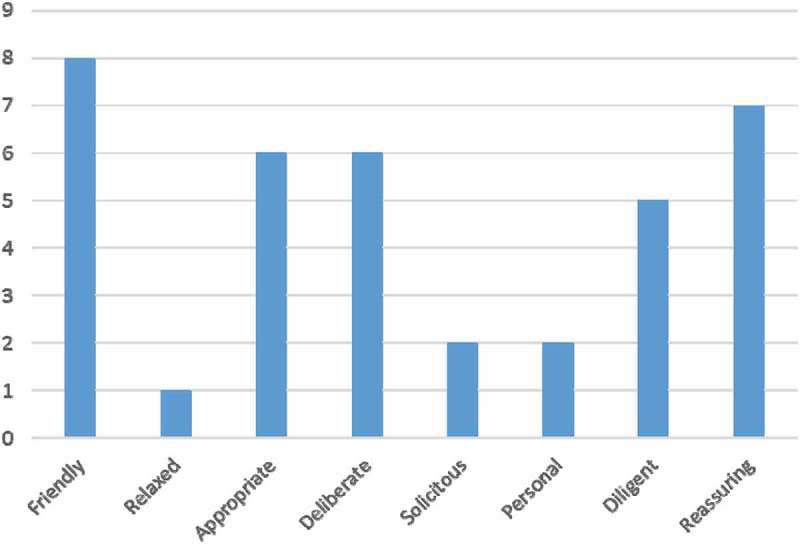
Patients’ perspective of the visit (*n* = 8).

None of the eight patients gave negative feedback after the visit. The adjectives to describe the atmosphere during the visit are presented in Figure 3.

## Discussion

Transforming health professionals´ education for the new century to strengthen health systems in an interdependent world is recommended by the Lancet commission.[10,20] The linkage between professional education, health conditions and service system is crucial, and there is a growing need to develop curricula of competencies to patient and population needs.[10,20] Health professionals’ education is criticised for being fragmented, and interprofessional teamwork skills and better communication are needed to improve patient safety and cost-effective qualitative care.[7] Training future health care providers to work in IP teams will improve the health care outcomes of the patients.[21]

The medical faculty of University of Oulu and Oulu University of Applied Sciences have previously carried out IP training sessions in the primary health care settings with encouraging results.[17,18] The special aspect of this study was the close collaboration between the educational organisations and organisations from the service system. The driving force towards the collaboration between the organisations has been the current Finnish health care reform, and the geographical proximity of the different organisations as well as enthusiasm of key persons made the IP training possible.

This trial was the first IPL experience in a clinical setting for both medical and nursing students involved in this study, and it was received with great enthusiasm, shared by the patients, teachers and local primary health care workers. The result was that students complemented each other well. They valued the mutual learning experience and felt comfortable with working together. This was seen in attitudes and readiness for IPE, which were scored high in RIPL scale and in pair work. The situation was challenging for undergraduate students, but working in an IP team encouraged the students to share knowledge and skills. The lowest scoring in RIPLS was in subscale “Roles and responsibilities”, where medical students scored significantly lower compared to nursing students. Medical students involved in the training were third-year students, thus they were in their first clinical year. Nursing students were more experienced in clinical skills. RIPL scale is validated and widely used, but the subscale “roles and responsibilities” has been criticised as having the lowest reliability.[8]

The primary care setting was unfamiliar to all students. Both student groups learned a lot about understanding the “big picture” of the holistic patient care, as well as time management, prioritisation and utilising all possible resources. Medves et al. [22] pointed out that learners from different programmes need varied opportunities to communicate and work together, thus also training in outpatient primary care setting is valuable. Furthermore Darlow et al. [23] found that even brief interventions improve attitudes for IPL.

Students performed well with team-based skills such as patient centredness, communication and team functioning. These competencies were defined valuable in Canadian IP competency model.[13] Paquette-Warren et al. [24] also highlighted the importance of team education to improve the care of chronic illnesses in primary health care. The patients described the visit with positive adjectives and felt they had received quality care. According to Barwell et al. [25] IPL increases the idea of each other’s roles, gives understanding of interpersonal skills and creates teams that work together better and improve patient experience. The link between practice and education systems is essential in order to build relevant IP competencies that students require.[16,26]

IP diabetes training was an innovate new model to organise IP training in the primary care sector, which is increasingly responsible for patients with major chronic public health issues. Collaboration with the different professionals in outpatient primary care and specialised hospital care as well as health care educators was highly valuable.

Most difficult challenges we encountered were administrative issues, for example the access to patient recording systems was time limited. Time management was challenging for the planning and implementing team too, but since all parties involved recognised the importance of IP sessions, despite the busy schedules mutual time slots were found for planning meetings.

The outpatient training periods of patients with diabetes type 2 have given valuable models to further organise and develop IPT in primary health care sector. Collaboration between different levels of care and health policies is important when developing health professionals’ education.[25] This IP teamwork experience benefits both future and current health care professionals to better organise the care of chronic illnesses.

## Conclusion

Following from the positive experience of this programme, as well as the enthusiastic student and patient feedback, other IP pilots have also begun. Starting from this autumn, IP diabetes type 2 outpatient primary care training will be organised for all medical students and a large proportion of nurse students. The faculty management support of both universities, and the support of management and professionals of Oulu City Health and Social Services and University Hospital of Oulu, have been essential in making these training sessions happen.

Team work has been emphasised as a key feature of Finnish health service reform. The city of Oulu has aimed to develop health and social care services in a more cost effective and client-centred direction. Doctors and nurses are in many instances working as pairs, and nurses are given more responsibility. This training was tailored to reflect this real life setting, and this model suits the principles of IPE well.
